# FER Regulated by miR-206 Promotes Hepatocellular Carcinoma Progression *via* NF-κB Signaling

**DOI:** 10.3389/fonc.2021.683878

**Published:** 2021-07-05

**Authors:** Wenzhou Ding, Ye Fan, Wenbo Jia, Xiongxiong Pan, Guoyong Han, Yao Zhang, Zhiqiang Chen, Yiwei Lu, Jinyi Wang, Jindao Wu, Xuehao Wang

**Affiliations:** ^1^ Hepatobiliary Center, The First Affiliated Hospital of Nanjing Medical University, Nanjing, China; ^2^ Key Laboratory of Liver Transplantation, Chinese Academy of Medical Sciences, National Health Commission (NHC) Key Laboratory of Living Donor Liver Transplantation (Nanjing Medical University), Nanjing, China; ^3^ Department of Anesthesiology, The First Affiliated Hospital of Nanjing Medical University, Nanjing, China

**Keywords:** FER, hepatocellular carcinoma, proliferation, metastasis, miR-206, NF-κB signaling, EMT

## Abstract

**Objectives:**

Feline sarcoma-related protein (FER) is known to play a critical regulatory role in several carcinomas. However, the exact biological function of FER in hepatocellular carcinoma (HCC) still needs to be investigated. The primary objective of this research was to investigate the unknown function and molecular mechanisms of FER in HCC.

**Materials and Methods:**

The expression level of FER in HCC tissue samples and cells was examined by RT-qPCR, immunohistochemistry and western blot. Cellular and animal experiments were used to explore the effect of FER on the proliferative and metastatic capacities of HCC cells. The crosstalk between FER and NF-κB signaling was explored by western blot. The upstream factors that regulate FER were evaluated through dual-luciferase experiments and western blot assays.

**Results:**

FER was overexpressed in HCC specimens and HCC cell lines. FER expression levels were positively associated with unfavorable clinicopathological characteristics. The higher the expression of FER was, the worse the overall survival of HCC patients was. The results of loss-of-function and gain-of-function experiments indicated that knockdown of FER decreased, while overexpression of FER increased, the proliferation, invasion and metastasis of HCC cells *in vitro* and *in vivo*. Mechanistically, we found that FER activated the NF-κB signaling pathway and stimulated epithelial-to-mesenchymal transition (EMT). We also found that FER was directly regulated by miR-206, and the downregulation of miR-206 was associated with proliferation and metastatic progression in HCC.

**Conclusions:**

The present research was the first to reveal that a decrease in miR-206 levels results in an increase in FER expression in HCC, leading to enhanced cell growth and metastatic abilities *via* activation of the NF-κB signaling pathway.

## Introduction

As a common malignant carcinoma, hepatocellular carcinoma (HCC) is ranked as the second leading cause of cancer-related mortality worldwide, particularly in China (1). During the past decade, despite the availability of advanced therapeutic options, the predominant curative options for HCC are still liver transplantation and surgical resection; however, the high frequency of tumor recurrence and metastasis following these therapeutic strategies is thought to be the primary reason for the poor prognosis of HCC patients (2). Hence, exploring the mechanism underlying HCC development and progression and identifying potential new therapeutic targets for HCC patients are urgently needed.

Feline sarcoma-related protein (FER) is a nonreceptor protein tyrosine kinase, that is localized in both the cytoplasm and nucleus of mammalian cells (3). FER is ubiquitously expressed, and it is structurally characterized by an F-BAR domain, an SH2 domain, and a COOH-terminal tyrosine kinase domain (4, 5). FER is overexpressed in various cancers, including pancreatic ductal adenocarcinomas (6), bladder urothelial cell carcinoma (7), melanoma (8), renal cell carcinoma (3), and breast cancer (9), and has been shown to enhance tumor cell metastasis and proliferation. However, the exact function of FER in HCC progression and the relevant biological molecular mechanism require further elucidation.

Our research has demonstrated the upregulation of FER in HCC clinical specimens and cell lines, and upregulation of FER was pathologically associated with poor prognosis. FER overexpression stimulated HCC cell growth and metastasis through stimulation of the NF-κB signaling pathway and induction of epithelial-to-mesenchymal transition (EMT). Furthermore, we discovered that FER was a direct regulatory target of miR-206, which was expressed at lower levels than usual in HCC. Upregulation of miR-206 attenuated the growth and migration of HCC cells. Moreover, the inhibition of FER by miR-206 could be partially reversed by the overexpression of FER. In conclusion, our findings revealed that FER acts as an oncogene and is possibly a promising research direction for the further investigation of therapeutic strategies for HCC.

## Materials and Methods

### HCC Patients and Tissues

The experiments conducted in this study were approved by the local Ethics Committee of The First Affiliated Hospital of Nanjing Medical University (Nanjing, China). The participants enrolled in this study all signed the informed consent form. HCC tissue samples and paracancerous normal samples were obtained from patients who underwent radical hepatectomy at The First Affiliated Hospital of Nanjing Medical University between October 2012 and December 2014. Confirmation of the HCC tissues were conducted through pathological examination, and the tissues were immediately stored by utilizing liquid nitrogen.

### HCC Cell Lines and Culture

Immortalized human hepatocyte L02, and the HCC cell lines SMMC7721, HepG2, MHCC97L, HCCLM3, Huh7 and Hep3B were purchased from the China Center for Type Culture Collection (Wuhan, China). YY8103 cells were obtained from the Key Laboratory on Living Donor Liver Transplantation. All the cell lines were cultured in Dulbecco’s modified Eagle’s medium (DMEM) (Invitrogen, Thermo Fisher Scientific, Inc., Carlsbad, CA, USA) supplemented with 10% fetal bovine serum (FBS) (Gibco, Life Technologies, Carlsbad, CA, USA), 50 U/ml penicillin (Invitrogen) and 50 U/ml streptomycin (Invitrogen) in a humidified 5% CO2 incubator at 37°C.

### RNA isolation and real-time quantitative polymerase chain reaction (RT-qPCR)

RNA was harvested from liver tissue samples and cell lines *via* TRIzol Reagent (Invitrogen), and the procedure followed the provided manual. cDNA synthesis was performed using a PrimeScript RT Reagent kit with gDNA Eraser (TakaRa, Dalian, China). We obtained bulge-loop™ miRNA RT-qPCR primer sets specific for miR-206, miR-199b-5p and U6 from RiboBio (Guangzhou, China). β-actin was used as the negative control for the mRNAs, U6 was selected as the internal control for the miRNAs, and the results were calculated according to the description of the 2^-ΔΔCT^ method (10). The primer sequences used in this research are listed in **Supporting Table 1**.

### Total Protein Acquisition and Western Blotting Examination

Phenylmethanesulfonyl fluoride (PMSF) (1mM) was added to the prechilled radioimmunoprecipitation assay (RIPA) buffer to obtain cell lysates, and these lysates were used to acquire the total proteins. The protein concentrations were quantified by a bicinchoninic acid (BCA) Protein Assay Kit (Beyotime, Nantong, China). Protein separation was conducted through sodium dodecyl sulfate-polyacrylamide gel electrophoresis (SDS-PAGE). Then, the proteins were transferred to a polyvinylidene fluoride (PVDF) membrane (Merck Millipore, Burlington, MA, USA). 5% skim milk in Tris-buffered saline-Tween (TBST) was used to block the membranes at ambient temperature for 2 h. Then, the membranes were incubated with diluted primary antibodies at 4°C overnight, followed by three washes with TBST. The membranes were incubated with horseradish peroxidase (HRP)-conjugated secondary antibodies for 2 h at 37°C, washed with TBST, and visualized by the Super ECL Detection Reagent (Yeasen, Shanghai, China). The protein density was then quantified using Image Lab software (Bio-Rad, Hercules, CA, USA). The antibodies used in this study are listed in **Supporting Table 2**.

### Immunohistochemistry

HCC tissues, adjacent normal tissues and subcutaneous tumors from nude mouse were fixed in 4% paraformaldehyde, and the samples were then embedded in paraffin blocks. The IHC methods and imaging protocols were conducted and the images were evaluated as previously described (11). The IHC intensity score was assigned as follows: 0 (negative), 1 (weak), 2 (medium), and 3 (strong). Positive cell proportions were scored as 0 (≤10%), 1 (10%-25%), 2 (26%–50%), 3 (51%–75%), and 4 (>75%). The final score was defined by multiplying the intensity score by the positive rate score.

### Transfection

Lentiviruses overexpressing FER, lentiviral-based small hairpin RNA (shRNA) against FER, miR-206 mimics and miR-206 inhibitor were purchased from GenePharma (Shanghai, China). Lentiviruses were used to infect HCC cells based on the manufacturer’s protocol. At 48 h after infection, the cells were incubated with puromycin (5 μg/mL) for two weeks to establish stable cell clones. The sequences are provided in **Supporting Table 1**.

### Cell Counting Kit-8 Assay

Cell proliferative capacity was measured by CCK8 assay (Dojindo Laboratories, Kumamoto, Japan) following the manufacturer’s description. Briefly, 1 × 10^3^ cells/well were seeded into 96-well plates with 100 μl of culture medium. After culturing for 24 h, 10μl CCK-8 solution was added to each well, and the cells were incubated at 37°C for 2 h. The whole procedure was performed in the dark. The absorbance was then measured at 450 nm wavelength with an ELISA plate reader. For the rescue experiments, the cells were pretreated with TNFα (10 ng/ml). Each experiment was repeated at least three times.

### Colony Formation Assay

Cells (5 × 10^2^ cells/well) were plated into six-well plates and incubated for 2 weeks. The colonies that formed were fixed in 4% paraformaldehyde and counted after 1% crystal violet staining. The numbers of the colonies were used to evaluate cell proliferation. The assay was analyzed in triplicate.

### Wound Healing Assay

To further explore the regulatory effect of FER on the migration of HCC cell lines, cells were seeded into 6-well plates. After reaching 90-95% confluence, wounds were introduced by scratching the surface of the plates using a 200 μL plastic pipette tip. In the rescue experiments, TNFα (10 ng/ml) was used to activate the NF-κB pathway. Wound closure was monitored and imaged at different time points (0 and 48 h) by an inverted microscope (Olympus, Tokyo, Japan). Photographs of three random microscopic fields across three replicate wells were captured for quantification analysis.

### Transwell Assays

Transwell chambers (8 μm pore size, Corning, NY, USA) were used to estimate the invasive and migratory abilities of HCC cells. For the cell migration assay, 2×10^4^ cells in 250 μL of serum-free DMEM with or without TNFα (10 ng/ml) were seeded into the upper chambers, while 750 μL DMEM containing 10% FBS was added to the lower chambers. To conduct the invasion assay, the upper chambers were precoated with 50 μL mixture of Matrigel (dilution, 1:8; BD Biosciences, Franklin Lakes, NJ, USA). A total of 5 × 10^4^ cells in 250 μL of serum-free DMEM with or without TNFα (10 ng/ml) were seeded into the upper chambers. The cells were cultured in 5% CO2 and 37°C for 48 h. Next, the nonmigrating or noninvading cells on the upper side of each insert were removed using a cotton tip. The transwell chambers were subsequently fixed in 4% phosphate-buffered neutral formalin for 30 min and stained with 1% crystal violet. Images of three random microscopic fields were captured for quantitative analysis. The experiments were replicated more than three times independent time.

### Luciferase Reporter Assay

The overlapping sequences of miR-206 in the FER 3’ UTR were predicted by miRDB, TargetScan and miRanda. Wild-type (WT) or mutant (MUT) FER-3’ UTR sequences were synthesized by Genescript (Nanjing, China) and inserted into the pGL3 plasmid (Ambion, Austin, TX, USA). The indicated cells were plated and cultured for 24 h. Then, the relevant plasmids were cotransfected with the miR-206 mimic or NC using Lipofectamine 3000 (Invitrogen). After 48 h, the relevant luciferase activity was determined using the Dual-Luciferase Reporter Assay System (Promega, USA) based on the description of the manufacturer.

### *In Vivo* Experiments

The animal experiments were carried out in accordance with the National Institutes of Health guidelines, the mice were housed in an aseptic environment, and the animal studies were approved by the local ethics committee of The First Affiliated Hospital of Nanjing Medical University. For the subcutaneous tumor model, 4-week-old male BALB/c nude mice were purchased from the Model Animal Research Center of Nanjing Medical University (Nanjing, China). A total of 5 × 10^6^ HCC cells were diluted in 100 μL PBS and subsequently injected into the flanks of nude mice. The tumor size was recorded ever three days. The following formula was used to calculate the volume of the tumor: [(length × width^2^)/2]. The mice were sacrificed 4 weeks later, and the tumor tissues were removed, measured and fixed. Sections were subjected to histopathological analysis. For the *in vivo* metastasis model, 6-week-old male BALB/c nude mice were injected with 1 × 10^6^ HCC cells *via* the tail vein. The mice were sacrificed after 8 weeks of feeding, and the lungs were harvested and fixed. Sections were subjected to H&E staining and analyzed for the presence of metastatic nodules.

### Statistical Analysis

Statistical analysis was performed using SPSS version 18.0 (Chicago, IL, USA) and GraphPad Prism version 7.0 software (La Jolla, CA, USA). Quantitative data were recorded as the mean ± standard deviation. Differences between two groups were evaluated using a two-tailed Student’s t-test. The chi-squared test was used to determine the relationship between FER expression and clinical parameters. Survival rates were plotted with the Kaplan-Meier estimator and log-rank analysis. A Spearman correlation test was used to analyze the association between miRNA and FER. p < 0.05 indicates statistically significant differences.

## Results

### Increased FER Expression Levels in HCC Are Associated With Poor Outcome

**Figure 1:**
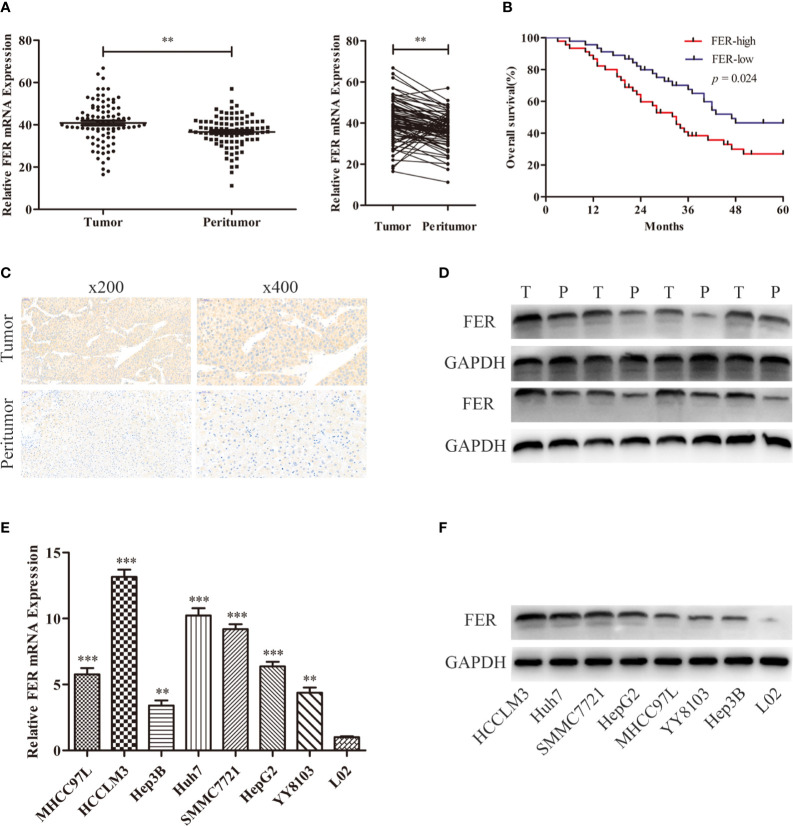
FER is upregulated in HCC and is correlated with poor clinical prognosis. **(A)** FER mRNA expression was analyzed in 90 pairs of HCC samples and the related paracancerous tissue samples by RT-qPCR. **(B)** The overall survival (OS) of patients with variable FER expression was assessed *via* Kaplan-Meier analyses. **(C)** The results of FER IHC staining in HCC tumor tissues and corresponding peritumor tissues. **(D)** The results of western blotting of FER in HCC tissues and peritumor tissues. **(E)** FER mRNA expression levels in HCC cell lines and the immortalized human hepatocyte L02 cell line. **(F)** Western blotting analysis of FER protein levels in HCC cell lines and normal hepatic L02 cell line. The data are presented as the mean ± SD of three independent experiments. (T, tumor; P, peritumor; **P < 0.01, ***P < 0.001).

To examine the FER expression levels in HCC samples and cell lines, RT-qPCR was used to assess its expression in 90 pairs of HCC samples and various HCC cell lines. The research results indicated that FER mRNA was upregulated in 76/90 (84.44%) HCC specimens compared with matched normal tissues (**Figure 1A**). Immunohistochemistry(IHC) and western blotting also verified the increased levels of FER in HCC tissues (**Figures 1C, D**). The clinicopathological analysis revealed the close association between FER expression and tumor multiplicity, tumor size, Edmondson stage, TNM stage and microvascular invasion (**Table 1**). According IHC score, HCC patients were divided into FER high expression group and FER low expression group, and survival analysis showed that FER high expression group exhibited decreased overall survival (OS) (**Figure 1B**). Next, we investigated the expression of FER in HCC cell lines by RT-qPCR and western blotting, and the results revealed that FER was overexpressed in all the studied HCC cell lines (HCCLM3, Huh7, SMMC7721, HepG2, 97L, YY8103 and Hep3B) compared with the normal liver cell line L02 (**Figures 1E, F**). The result described above suggested that FER is upregulated in HCC, and upregulated FER is associated with poor prognosis and might serve as a novel and promising target for mediating the progression of HCC.

**Table 1:** Correlation between FER expression and clinicopathological features.

Clinicopathological features		All cases	FER		*p* value
			High expression	Low expression	
Age (years)	<60	63	30	33	0.490
	≤60	27	15	12	
Gender	Female	23	14	9	0.227
	Male	67	31	36	
HBV	Negative	11	4	7	0.334
	Positive	79	41	38	
Tumor multiplicity	Single	60	25	35	**0.025**
	Multiple	30	20	10	
Tumor size (cm)	≤5	51	20	31	**0.019**
	<5	39	25	14	
α-fetoprotein (ng/ml)	≤200	32	14	18	0.378
	<200	58	31	27	
Edmondson stage	I-II	59	25	34	**0.046**
	III-IV	31	20	11	
TNM stage	I	36	13	23	**0.031**
	II-III	54	32	22	
Microvascular invasion	Yes	23	16	7	**0.030**
	No	67	29	38	

HBV, hepatitis B virus; TNM, tumor-node-metastasis.

The bold number means statistically significant.

### Decreased FER Expression Attenuates the Proliferative, Migratory, and Invasive Capacities of HCC Cells

**Figure 2:**
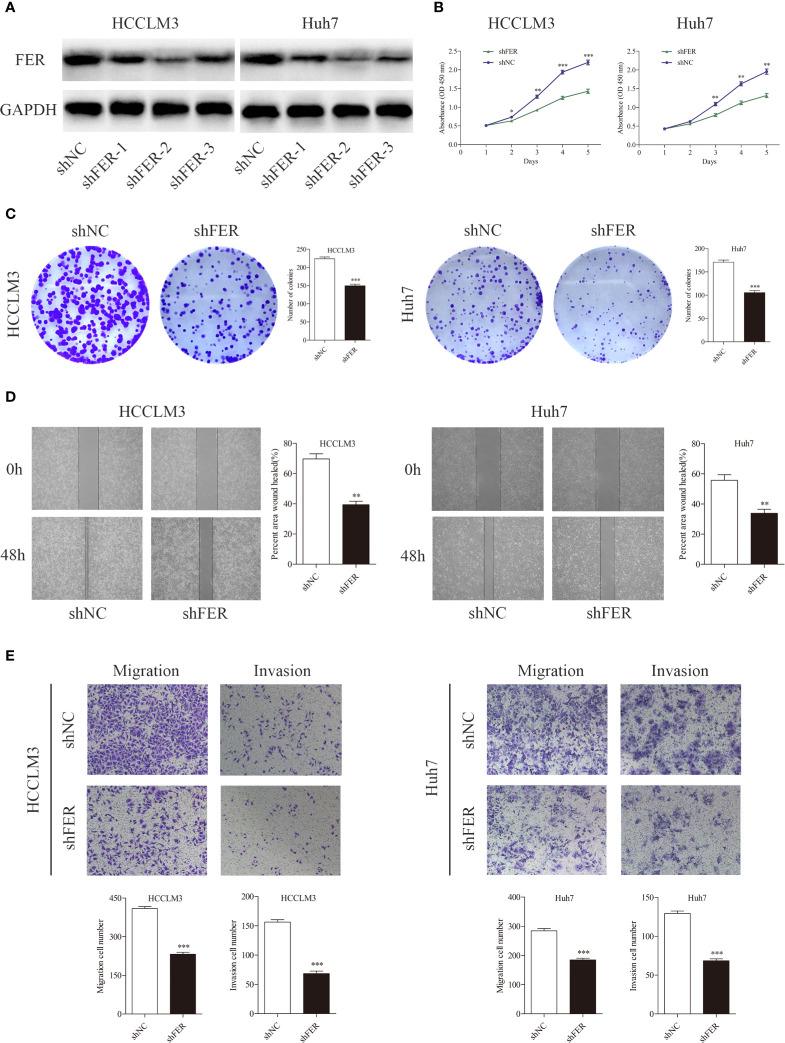
Downregulation of FER attenuates the proliferative and metastatic abilities of HCC cells in *vitro*. **(A)** The western blot results show the FER levels after lentiviral-based small hairpin RNA (shRNA) transfection. **(B)** Growth curves based on CCK-8 assay results of HCCLM3 and Huh7 cells with FER silencing. **(C)** Representative images of colony formation assay results of HCCLM3 and Huh7 cells subjected to different treatments. **(D)** Knockdown of FER in HCCLM3 and Huh7 cells inhibited cell motility, as assessed by wound healing assay. **(E)** Transwell assays showed that HCC cells transfected with shFER exhibited reduced invasion and migration abilities. (*P < 0.05, **P < 0.01, ***P < 0.001).

Based on the level of FER expression in the HCC cell lines, lentiviruses carrying short hairpin RNA (shRNA) or negative control (shNC) were transfected into the HCCLM3 and Huh7 cell lines, which exhibited higher FER expression than the other cell lines. The FER knockdown efficiency in these cells was confirmed by western blotting. shFER-2 exhibited the best knockdown efficiency and was used in the subsequent experiments (**Figure 2A**). CCK-8 and colony formation assays were carried out to assess HCC cell proliferation. The assays indicated that downregulation of FER inhibited the growth and foci formation of the HCCLM3 and Huh7 cells (**Figures 2B, C**). In the wound healing assay and Transwell assays, both the HCCLM3 and Huh7 cells transfected with shFER exhibited delayed wound healing, and suppressed cell migration and invasion (**Figures 2D, E**). The results described above indicate that downregulation of FER could attenuate the cell growth, migration, and invasive capacities of HCC cells *in vitro*.

### Overexpression of FER Promotes HCC Cell Growth, Migration, and Invasion

**Figure 3:**
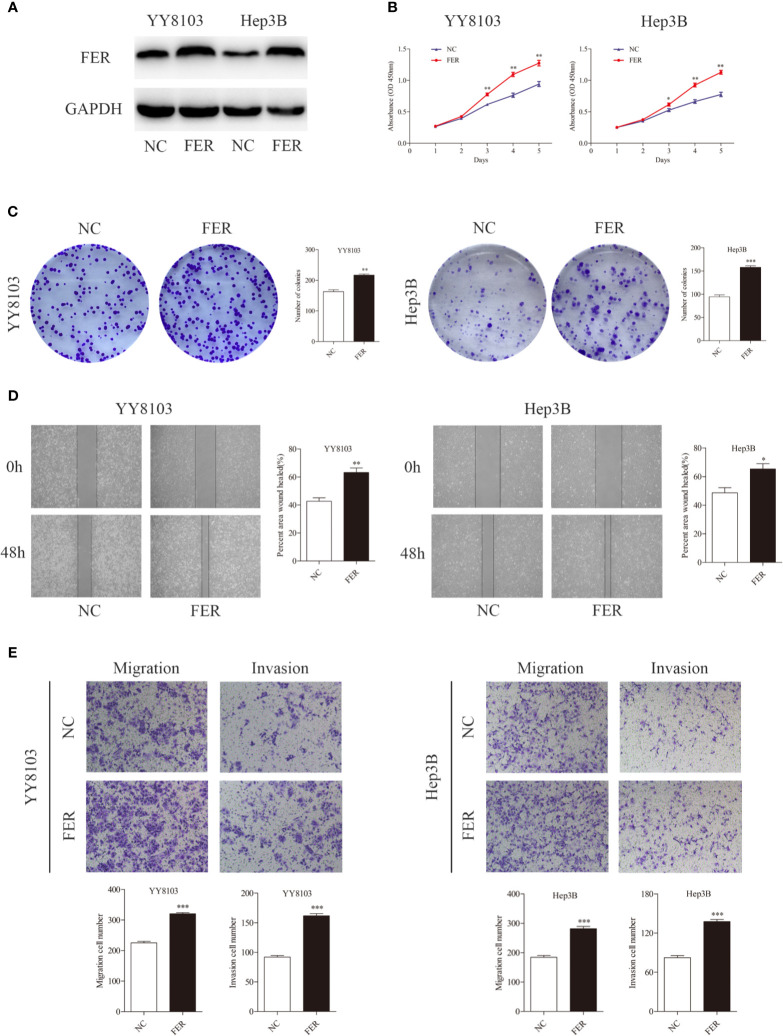
Upregulation of FER augments cell growth and metastasis in *vitro*. **(A)** FER expression levels were explored in YY8103 and Hep3B cells transfected with FER overexpression lentivirus. **(B)** CCK-8 assay results of YY8103 and Hep3B cells. **(C)** Colony formation assay results of YY8103 and Hep3B cells overexpressing FER. **(D)** Wound healing assay suggested that upregulation of FER increased motility in YY8103 and Hep3B cell lines. **(E)** Transwell assays demonstrated that HCC cells overexpressing FER showed increased cell invasion and migration. (*P < 0.05, **P < 0.01, ***P < 0.001).

We performed gain-of-function experiments by overexpressing FER in the YY8103 and Hep3B cell (low FER expression) using lentivirus. Western blotting confirmed the efficiency of FER overexpression (**Figure 3A**). The CCK-8 and colony formation assays revealed that upregulation of FER enhanced the proliferation of the YY8103 and Hep3B cells (**Figures 3B, C**). Moreover, the metastatic and invasive abilities of the cells were assessed by wound healing and transwell chamber assays. The results demonstrated that FER overexpression facilitated cell migration and invasion in the YY8103 and Hep3B cell lines (**Figures 3D, E**). Taken together, these data were consistent with the FER downregulation data and demonstrated that FER is an oncogene in HCC *in vitro*.

### FER Overexpression Augments the Proliferation and Metastasis of HCC Cells *In Vivo*

**Figure 4:**
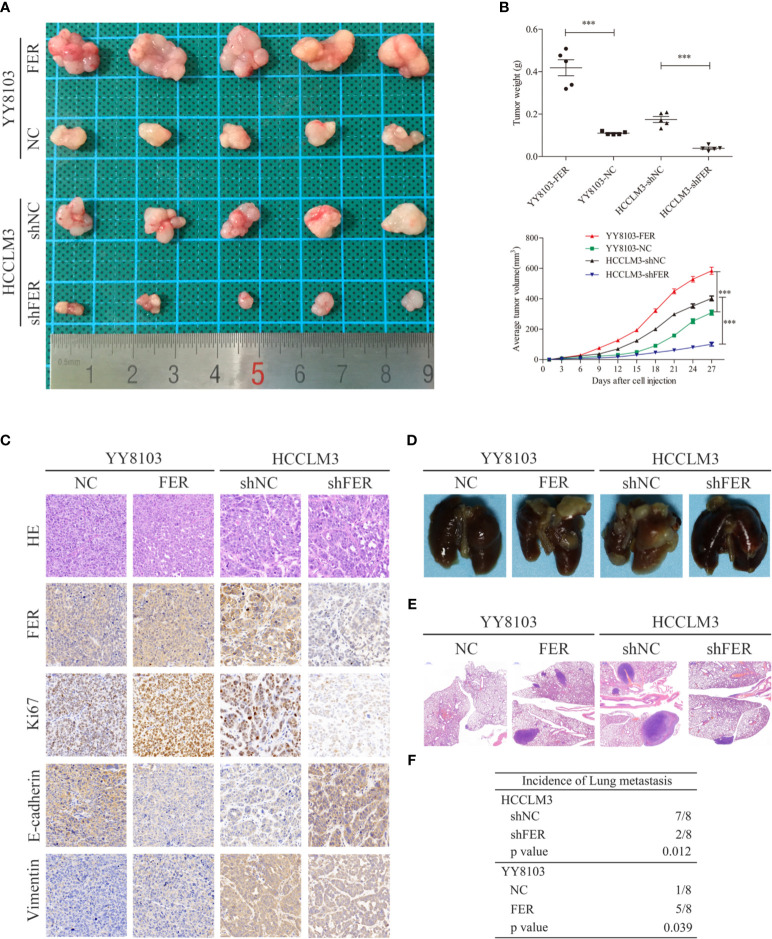
FER increase HCC growth and metastasis in *vivo*. **(A)** Representative images of subcutaneous tumor tissues derived from the indicated HCC cells. **(B)** Tumor weight and volume from the four groups were recorded and analyzed. **(C)** Representative images of HE staining and immunohistochemical analysis of the FER, Ki-67, E-cadherin and Vimentin protein levels in the subcutaneous tumor tissues from four groups (400x). **(D)** Representative images of the indicated groups of the pulmonary metastasis model. **(E)** Representative HE staining images of lung tissues from the four groups (20x). **(F)** The incidence of pulmonary metastasis in the four groups was statistically analyzed. (***P < 0.001).

To further explore the oncogenic function of FER *in vivo*, we established subcutaneous tumor model and pulmonary metastasis mouse models *via* tail vein injection. As shown in the subcutaneous tumor model, FER overexpression increased, while FER knockdown decreased tumor growth in nude mice, as reflected by the tumor weight and volume (**Figures 4A, B**). HE, FER, Ki-67, E-cadherin and Vimentin staining of the xenograft tumors was conducted, and the results indicated that FER, Ki-67 and Vimentin expression was significantly increased and E-cadherin expression was decreased in the YY8103-FER group, whereas the opposite results were obtained in the HCCLM3-shFER group (**Figure 4C**). These data illustrated that FER stimulated HCC growth and EMT *in vivo*.

In addition, a mouse model of pulmonary metastasis *via* tail vein injection was established to examine the functional role of FER *in vivo*. Consistent with our discovery *in vitro*, compared to the control, knockdown of FER attenuated the lung metastasis of HCCLM3 cells. Conversely, the lung metastases in the YY8103-FER group were increased compared with those in the YY8103-NC group (**Figure 4D**). Lung tissue samples were subjected to H&E staining and analyzed for the presence of metastatic nodules; the data provided further evidence that FER promoted HCC cell metastasis *in vivo* (**Figures 4E, F**). Thus, our results demonstrated that FER facilitated HCC growth and migration *in vivo*.

### FER Activates NF-κB Signaling and Induces the Epithelial-Mesenchymal Transition Process in HCC Cells

**Figure 5:**
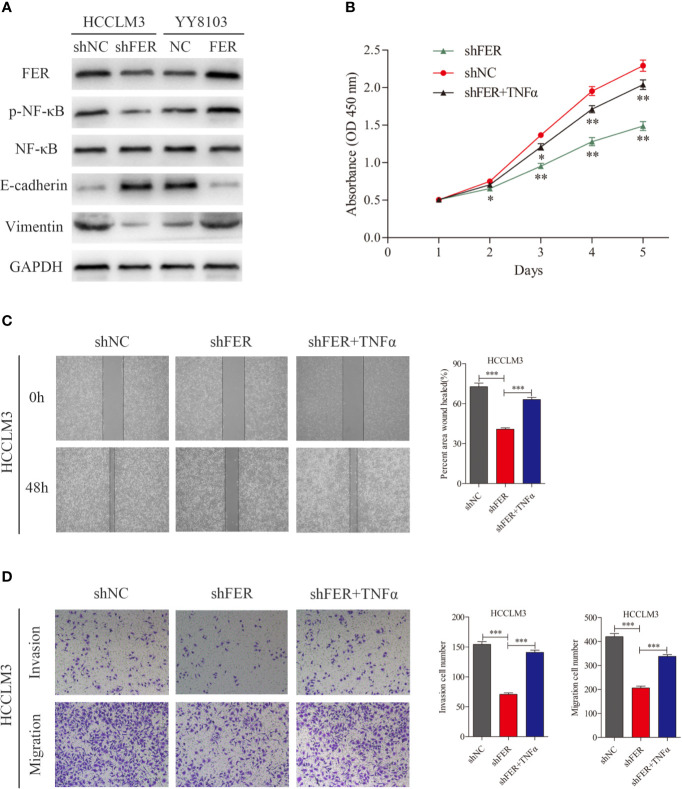
FER facilitates HCC progression by activating NF-κB signaling and inducing EMT. **(A)** Proteins expression were measured by western blot in YY8103 and HCCLM3 cells. **(B)** CCK-8 assay was conducted to investigate the proliferation of HCCLM3-shFER cells treated with TNFα. **(C)** Wound healing assay was performed to evaluate the motility of HCCLM3-shFER cells treated with TNFα. **(D)** Cell invasion and migration in the indicated groups were detected by transwell assays. (*P < 0.05, **P < 0.01, ***P < 0.001).

Prior studies have observed that FER is activated by cell-surface receptors, such as EGFR and PDGFR (12). We investigated the related signaling cascades. The western blotting data showed that FER knockdown in HCCLM3 cells decreased the levels of phosphorylated NF-κB, and the mesenchymal marker vimentin and increased the levels of the epithelial marker E-cadherin, whereas FER overexpression in YY8103 cells exerted the opposite effects (**Figure 5A**). During EMT, tumor-associated epithelial cells lose their epithelial phenotype and acquire mesenchymal features, and this transition increases tumor cell invasiveness and motility. To further elucidate whether FER activates NF-κB signaling, TNFα, an NF-κB activator, was added to the HCCLM3-shFER cells. CCK-8, wound healing and Transwell assays revealed that NF-κB activation reversed the decreased proliferation, invasion and migration observed in HCCLM3 cells with downregulated FER (**Figures 5B–D**). Taken together, the results suggest that FER promotes HCC progression by activating NF-κB signaling.

### FER Serves as a Direct Target Gene of miR-206 in HCC

**Figure 6:**
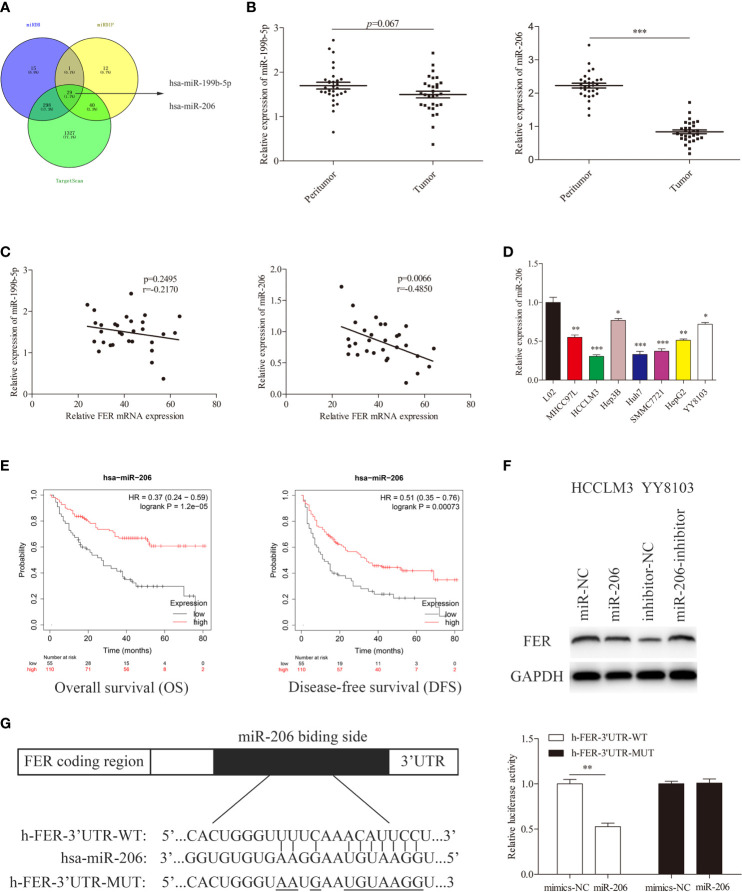
miR-206 directly targets FER in HCC cells. **(A)** Venn diagram showing miR-206 and miR-199b-5p computationally predicted to target FER by three databases. **(B)** Relative miRNA expression levels in 30 pairs of HCC tissues were determined using qRT-PCR. **(C)** Spearman correlation analysis revealed the association between FER and candidate miRNAs in the 30 HCC samples. **(D)** The expression level of miR-206 in HCC cell lines and the L02 cell line. **(E)** The overall survival (OS) and disease-free survival (DFS) of HCC patients with different miR-206 expression levels (data from Kaplan-Meier Plotter). **(F)** The expression level of FER was analyzed in HCCLM3 cells transfected with the miR-206 mimic or miR-NC and YY8103 cells transfected with the miR-206 inhibitor or inhibitor NC. **(G)** The predicted miR-206 targeting sequence in the FER-3’ UTR (FER-3’ UTR-WT), and luciferase activity, as detected in a luciferase reporter assay. (*P < 0.05, **P < 0.01, ***P < 0.001).

MiRNAs have been shown to regulate more than one-third of human genes and play a significant role in tumor regulation (13). To further characterize the upstream regulator of FER, we searched several publicly available databases, including miRDB, miRDIP and TargetScan, and identified candidate miRNAs (miR-199b-5p and miR-206) (**Figure 6A**). We first measured the expression levels of these miRNA in HCC tissues using RT-qPCR. The results demonstrated that only miR-206 expression was decreased in our HCC samples, and Spearman correlation analysis revealed that FER expression was negatively associated with miR-206 expression but not miR-199b-5p expression, in 30 pairs of HCC tissues (**Figures 6B, C**). The same trend in the variation of miR-206 was observed in HCC cells compared with L02 cells (**Figure 6D**). Data from a public database (Kaplan-Meier Plotter) showed that high miR-206 expression was associated with better overall survival and disease-free survival (**Figure 6E**). Western blot analysis verified that the overexpression of miR-206 decreased the level of FER in HCCLM3 cells, whereas miR-206 inhibition upregulated FER expression in YY8103 cells (**Figure 6F**). Finally, a dual-luciferase reporter assay was conducted to confirm that miR-206 can directly bind to FER. The wild-type (WT) or mutant (MUT) FER-3’ UTR was inserted into a reporter vector. The luciferase activity was significantly decreased in the HCCLM3 cells cotransfected with FER-3’ UTR-WT and miR-206, but it was almost unchanged in the cells cotransfected with FER-3’ UTR-MUT and miR-206 (**Figure 6G**). Overall, our studies demonstrate that FER can be negatively and directly regulated by miR-206.

### MiR-206 Inhibits HCC Cell Growth and Metastasis by Targeting FER

**Figure 7:**
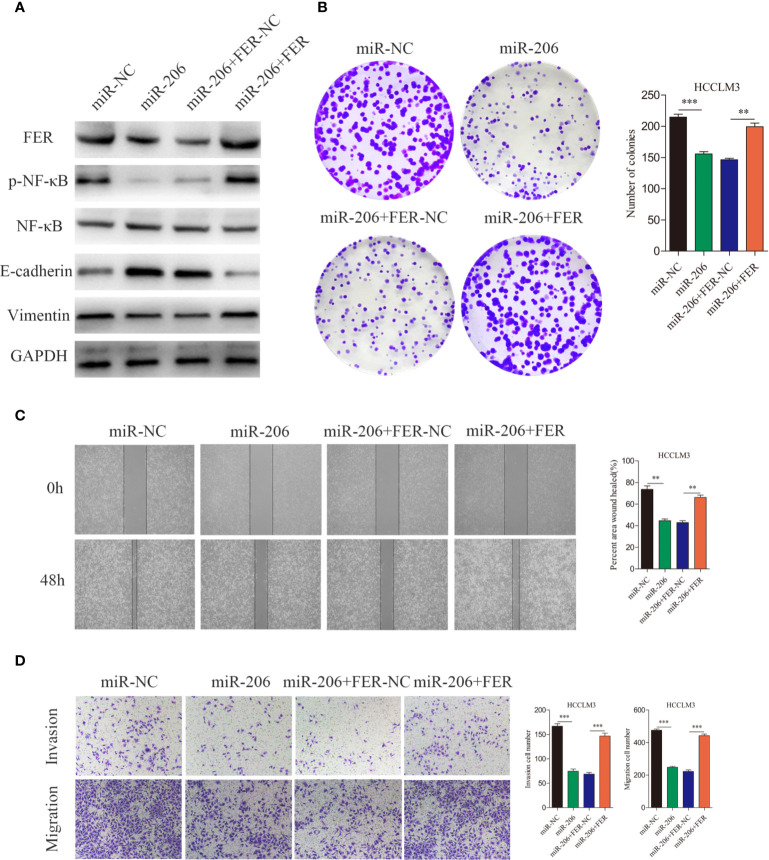
Rescue experiments were used to confirm that miR-206 is an upstream regulator of FER. **(A)** Western blotting analyses were used to analyze the FER overexpression-induced changes in the p-NF-κB, NF-κB, E-cadherin and vimentin expression levels in HCCLM3 cells treated with miR-206 mimic. **(B)** The colony formation assay was performed to determine the colony forming ability of the indicated groups. **(C)** Wound healing assays showed that FER overexpression restored the attenuated cell migration in HCCLM3 cells overexpressing miR-206. **(D)** The cell invasion and migration abilities of HCCLM3 cells treated with different lentiviruses were evaluated by transwell assays. (**P < 0.01, ***P < 0.001).

To further investigate whether miR-206 regulates HCC proliferation and metastasis by regulating FER expression, four groups of HCCLM3 cells were established. The western blotting results indicated that miR-206 overexpression decreased the levels of FER, p-NF-κB and vimentin and increased the levels of E-cadherin, while, FER upregulation attenuated these effects of miR-206 (**Figure 7A**). As shown in **Figure 7B**, the colony formation assay demonstrated that miR-206 overexpression inhibited cell growth, whereas FER rescued the proliferative capacity of the HCCLM3 cells. Wound healing assays and transwell assays were conducted to monitor the invasive and migratory abilities of the HCCLM3 cells. Upregulation of miR-206 decreased the migration and invasion abilities of the HCC cells, but overexpression of FER restored these abilities (**Figures 7C, D**). Collectively, the evidence described above further illustrates that FER is negatively regulated by miR-206 in HCC and that miR-206 inhibits HCC proliferation and metastasis.

## Discussion

Due to inadequate early specific symptoms, effective therapies and prognostic assessments, the survival rate of HCC patients is still poor. Hence, the pathological mechanism of HCC occurrence and development has always been a focus of research, and this research is expected to yield new therapeutic targets and diagnostic markers. In the current research, we showed that FER was overexpressed in HCC tissues and HCC cell lines, and a high expression level of FER was associated with poor overall survival in HCC patients. In vitro and *in vivo* functional experiments demonstrated that FER promoted cell growth and metastasis by regulating NF-κB/EMT signaling and was negatively regulated by miR-206 in HCC cells. Our results suggest that FER and miR-206 play pivotal roles in the course of HCC.

Previous studies revealed that FER was involved in a series of cellular processes, including the cell cycle, growth and metastasis (5, 14). For example, Ahn et al. reported that FER was involved in EGFR-induced cell migration and invasion in lung adenocarcinoma (15). Oneyama et al. found that FER was activated by the EGFR-c-Src axis and was associated with the progression of colon cancer (16). Hu et al. revealed that FER was associated with the migration, invasion, viability and apoptosis of bladder cancer (7, 17). Based on LC-MS/MS, Li et al. explored the tyrosine-phosphorylated protein expression profile in highly metastatic and nonmetastatic HCC cell lines (MHCC97H and Hep3B), and the study confirmed that FER was upregulated in highly metastatic HCC cells (18). Nevertheless, the specific biological function and pathological mechanism of FER in HCC have not been further illustrated. Consistent with previous studies, our work was the first to show that FER was overexpressed in HCC and promoted HCC cell proliferation and metastasis both *in vitro* and *in vivo*.

FER is usually activated downstream of certain growth factor receptors, such as PDGFR, HGFR and EGFR (19–21). However, the underlying molecular mechanisms are still unclear. It was reported that FER regulated GAB1 and MET phosphorylation and activated SHP2-ERK signaling in ovarian cancer (22). Downregulation of FER inactivated STAT3 in colon carcinoma cells (23). Interestingly, Guo et al. discovered that overexpression of FER also increased the phosphorylation of EGFR and ERK, which led to the activation of NF-κB (21). NF-κB signaling is of great importance for the induction and maintenance of MET, which promotes tumor metastasis in the early stages (24). Some cytokines, such as TNFα, IL-1 and EGF, can drive NF-κB activation (25). NF-κB activation inhibits apoptosis and promotes proliferation, invasion and inflammation in a variety of tumors (26, 27). Therefore, we explored the relationship between FER and NF-κB in HCC. In our study, FER overexpression promoted EMT by upregulating the p-NF-κB protein levels, but the total NF-κB level was not significantly changed.

To date, few studies have focused on the mechanisms by which FER is overexpressed in tumors. Chen et al. reported that the transcription factor YY1 directly binds to the promoter region of FER to regulate FER expression (6). MiRNAs have long been known to act as posttranscriptional regulators and to regulate ~1/3 of human coding genes (28). Based on publicly available databases, we found that miR-206 might be a potential upstream regulator of FER. MiR-206 was found to act as a tumor suppressor gene in multifarious tumors, including HCC (29–33). Here, we identified miR-206 as an upstream regulator of FER that directly targets FER in HCC cells.

In conclusion, our study was the first to demonstrate that FER acts as an oncogene that enhances HCC proliferation and metastasis both *in vitro* and *in vivo*. FER overexpression activates the NF-κB signaling pathway and promotes EMT. Moreover, FER is directly and negatively regulated by miR-206. Our discovery provides a new perspective for further investigating the mechanism of HCC. Targeting the FER signaling pathway may improve the treatment effects and prognosis of patients with HCC.

## Data Availability Statement

The original contributions presented in the study are included in the article/**Supplementary Material**. Further inquiries can be directed to the corresponding authors.

## Ethics Statement

The studies involving human participants were reviewed and approved by The ethics committee of The First Affiliated Hospital of Nanjing Medical University. The patients/participants provided their written informed consent to participate in this study. The animal study was reviewed and approved by The ethics committee of The First Affiliated Hospital of Nanjing Medical University.

## Author Contributions

WD, YF, and WJ conducted the experiments and wrote this manuscript. GH, YZ, ZC, YL and JYW conducted parts of the experiments. XP and JD analyzed the data. XW and JDW conceived and supervised this study. All authors contributed to the article and approved the submitted version.

## Funding

This work was supported by research grants from the Major Program of the National Natural Science Foundation of China (81530048 and 31930020), the National Natural Science Foundation of China (81870488), and the Southeast University and Nanjing Medical University Cooperative Research Project, China (2019DN0003).

## Conflict of Interest

The authors declare that the research was conducted in the absence of any commercial or financial relationships that could be construed as a potential conflict of interest.

## Abbreviations

FER, Feline sarcoma-related protein; HCC, hepatocellular carcinoma; EMT, epithelial-to-mesenchymal transition; DMEM, Dulbecco’s modified Eagle’s medium; FBS, fetal bovine serum; RT-qPCR, real-time quantitative polymerase chain reaction; PMSF, phenylmethanesulfonyl fluoride; PVDF, polyvinylidene fluoride; IHC, Immunohistochemistry; CCK8, Cell Counting Kit-8; TNM, tumor-node-metastasis; HE, Hematoxylin and eosin.

## References

[1] Bray F Ferlay J Soerjomataram I Siegel RL Torre LA Jemal A. Global Cancer Statistics 2018: GLOBOCAN Estimates of Incidence and Mortality Worldwide for 36 Cancers in 185 Countries. CA Cancer J Clin (2018) 68(6):394–424. 10.3322/caac.21492 30207593

[2] Serper M Taddei TH Mehta R D'Addeo K Dai F Aytaman A. Association of Provider Specialty and Multi-Disciplinary Care With Hepatocellular Carcinoma Treatment and Mortality. Gastroenterology (2017) 152(8):1954–64. 10.1053/j.gastro.2017.02.040 PMC5664153 28283421

[3] Miyata Y Kanda S Sakai H Greer PA. Feline Sarcoma-Related Protein Expression Correlates With Malignant Aggressiveness and Poor Prognosis in Renal Cell Carcinoma. Cancer Sci (2013) 104(6):681–6. 10.1111/cas.12140 PMC7657252 23445469

[4] Heath RJW Insall RH. F-BAR Domains: Multifunctional Regulators of Membrane Curvature. J Cell Sci (2008) 121:1951–4. 10.1242/jcs.023895 18525024

[5] Greer P. Closing in on the Biological Functions of Fps/Fes. Nat Rev Mol Cell Biol (2002) 3(4):278–89. 10.1038/nrm783 11994747

[6] Chen Q Zhang JJ Ge WL Chen L Yuan H Meng LD. YY1 Inhibitors the Migration and Invasion of Pancreatic Ductal Adenocarcinoma by Downregulating the FER/STAT3/MMP2 Signaling Pathway. Cancer Lett (2019) 463:37–49. 10.1016/j.canlet.2019.07.019 31404611

[7] Hu XD Zhang ZQ Liang ZF Xie D Zhang T Yu D. Downregulation of Feline Sarcoma-Related Protein Inhibits Cell Migration, Invasion and Epithelial-Mesenchymal Transition *via* the ERK/AP-1 Pathway in Bladder Urothelial Cell Carcinoma. Oncol Lett (2017) 13(2):686–94. 10.3892/ol.2016.5459 PMC5351348 28356947

[8] Ivanova IA Arulanantham S Barr K Cepeda M Parkins KM Hamilton AM. Targeting FER Kinase Inhibits Melanoma Growth and Metastasis. Cancers (2019) 11(3):419. 10.3390/cancers11030419 PMC6468679 30909648

[9] Ivanova IA Vermeulen JF Ercan C Houthuijzen JM Saig FA Vlug EJ. FER Kinase Promotes Breast Cancer Metastasis by Regulating α6- and β1-Integrin-Dependent Cell Adhesion and Anoikis Resistance. Oncogene (2013) 32(50):5582–92. 10.1038/onc.2013.277 PMC3898493 23873028

[10] Livak KJ Schmittgen TD. Analysis of Relative Gene Expression Data Using Real-Time Quantitative PCR and the 2^–ΔΔct^ Method. Methods (2001) 25(4):402–8. 10.1006/meth.2001.1262 11846609

[11] Chen ZQ Gao W Pu Ly Zhang L Han G Zuo X. PRDM8 Exhibits Antitumor Activities Toward Hepatocellular Carcinoma by Targeting NAP1L1. Hepatology (2018) 68(3):994–1009. 10.1002/hep.29890 29572888

[12] Kim L Wong TW. Growth Factor-Dependent Phosphorylation of the Actin-Binding Protein Cortactin is Mediated by the Cytoplasmic Tyrosine Kinase FER. J Biol Chem (1998) 273(36):23542–8. 10.1074/jbc.273.36.23542 9722593

[13] Berindan-Neagoe L Calin GA. Molecular Pathways: microRNAs, Cancer Cells, and Microenvironment. Clin Cancer Res (2014) 20(24):6247–53. 10.1158/1078-0432.CCR-13-2500 PMC4461223 25512634

[14] Li P Ma ZW Yu Y Hu X Zhou Y Song H. FER Promotes Cell Migration *via* Regulating JNK Activity. Cell Prolif (2019) 51(5):e12656. 10.1111/cpr.12656 PMC6797522 31264309

[15] Ahn J Truesdell P Meens J Kadish C Yang X Boag AH. Fer Protein-Tyrosine Promotes Lung Adenocarcinoma Cell Invasion and Tumor Metastasis. Mol Cancer Res (2013) 11(8):952–63. 10.1158/1541-7786.MCR-13-0003-T 23699534

[16] Oneyama C Yoshikawa Y Ninomiya Y Iino T Tsukita S Okada M. Fer Tyrosine Kinase Oligomer Mediates and Amplifies Src-Induced Tumor Progression. Oncogene (2016) 35(4):501–12. 10.1038/onc.2015.110 25867068

[17] Hu XD Guo ZW Xu JF Mei X Bi M Jiang F. Role of Feline Sarcoma-Related Protein in the Viability and Apoptosis of Bladder Cancer Cells. Mol Med Rep (2019) 19(6):5219–26. 10.3892/mmr.2019.10204 31059042

[18] Li HY Ren ZG Kang XN Zhang L Li X Wang Y. Identification of Tyrosine-Phosphorylated Proteins Associated With Metastasis and Functional Analysis of FER in Human Hepatocellular Carcinoma Cells. BMC Cancer (2009) 9:366. 10.1186/1471-2407-9-366 19835603 PMC2770568

[19] Lennartsson J Ma H Wardega P Pelka K Engström U Hellberg C. The Fer Tyrosine Kinase Is Important for Platelet-Derived Growth Factor-BB-Induced Signal Transducer and Activator of Transcription 3(STAT3) Protein Phosphorylation, Colony Formation in Soft Agar, and Tumor Growth In Vivo. J Biol Chem (2013) 288(22):15736–44. 10.1074/jbc.M113.476424 PMC3668732 23589302

[20] Fan GF Nicholas N. FER Mediated HGF-Independent Regulation of HGFR/EMT Activates RAC1-PAK1 Pathway to Potentiate Metastasis in Ovarian Cancer. Small GTPases (2020) 11(3):155–9. 10.1080/21541248.2017.1379931 PMC7549699 29099290

[21] Guo CH Stark GR. FER Tyrosine Kinase (FER) Overexpression Mediates Resistance to Quinacrine Through EGF-Dependent Activation of NF-kappaB. Proc Natl Acad Sci USA (2011) 108(19):7968–73. 10.1073/pnas.1105369108 PMC3093511 21518868

[22] Fan GF Zhang SW Gao Y Greer PA Tonks NK. HGF-Independent Regulation of MET and GAB1 by Nonreceptor Tyrosine Kinase FER Potentiates Metastasis in Ovarian Cancer. Genes Dev (2016) 30(13):1542–57. 10.1101/gad.284166.116 PMC4949327 27401557

[23] Orlovsky K Theodor L Malovani H Chowers Y Nir U. Gamma Interferon Down-Regulates Fer and Induces Its Association With Inactive Stat3 in Colon Carcinoma Cells. Oncogene (2002) 21(32):4997–5001. 10.1038/sj.onc.1205624 12118379

[24] Ren D Yang Q Dai YH Guo W Du H Song L. Oncogenic miR-210-3p Promotes Prostate Cancer Cell EMT and Bone Metastasis *via* NF-κB Signaling Pathway. Mol Cancer (2017) 16(1):117. 10.1186/s12943-017-0688-6 28693582 PMC5504657

[25] Hayden MS Ghosh S. Signaling to NF-Kappab. Genes Dev (2004) 18(18):2195–224. 10.1101/gad.1228704 15371334

[26] Karin M Greten FR. NF-kappaB: Linking Inflammation and Immunity to Cancer Development and Progression. Nat Rev Immunol (2005) 5(10):749–59. 10.1038/nri1703 16175180

[27] Wang L Niu ZD Wang X Li Z Liu Y Luo F. PHD2 Exerts Anti-Cancer and Anti-Inflammatory Effects in Colon Cancer Xenografts Mice *via* Attenuating NF-κB Activity. Life Sci (2020) 242:117167. 10.1016/j.lfs.2019.117167 31838134

[28] Bartel DP. MicroRNAs: Target Recognition and Regulatory Functions. Cell (2009) 136(2):215–33. 10.1016/j.cell.2009.01.002 PMC3794896 19167326

[29] Wang Y Xu HT Si LH Li Q Zhu X Yu T. MiR-206 Inhibits Proliferation and Migration of Prostate Cancer Cells by Targeting CXCL11. Prostate (2018) 78(7):479–90. 10.1002/pros.23468 29542173

[30] Wang P Gu JL Wang KJ Shang J Wang W. miR-206 Inhibits Thyroid Cancer Proliferation and Invasion by Targeting RAP1B. J Cell Biochem (2019) 120(11):18927–36. 10.1002/jcb.29213 31245877

[31] Samaeekia R Adorno-Cruz V Bockhorn J Chang YF Huang S Prat A. miR-206 Inhibits Stemness and Metastasis of Breast Cancer by Targeting MKL1/IL11 Pathway. Clin Cancer Res (2017) 23(4):1091–103. 10.1158/1078-0432.CCR-16-0943 PMC5247402 27435395

[32] Dai CX Xie YY Zhuang XP Yuan Z. MiR-206 Inhibits Epithelial Ovarian Cancer Cells Growth and Invasion *via* Blocking C-Met/AKT/mTOR Signaling Pathway. BioMed Pharmacother (2018) 104:763–70. 10.1016/j.biopha.2018.05.077 29807226

[33] Pang C Huang G Luo KL Dong Y He F Du G. miR-206 Inhibits the Growth of Hepatocellular Carcinoma Cells *via* Targeting CDK9. Cancer Med (2017) 6(10):2398–409. 10.1002/cam4.1188 PMC5633544 28940993

