# Supplementary material for: FER Regulated by miR-206 Promotes Hepatocellular Carcinoma Progression via NF-κB Signaling
Source: Front Oncol. 2021 Jul 5;11:683878. doi: 10.3389/fonc.2021.683878 (PMC8289706; doi:10.3389/fonc.2021.683878)
Supplement: Supplementary file 1 [file Table_1.docx]

**Supporting Table 1**. Primers and shFER and miR-206 used in the present study.

| Description | | Name | | Sequence |
| --- | --- | --- | --- | --- |
| Primers for qRT-PCR | | FER forward  FER reverse  β-actin forward  β-actin reverse | | 5′‐TTCGAGGGCACTGGGTTTTC‐3′  5′‐TTCCCTTGCCCAGTAATTCTCC‐3′  5′‐AAAGCGGCTGTTAGTCACTGG‐3′  5′‐GGGGCAGTTATTGCACTTGTC‐3′ |
| Sequence of shRNA |  | | shFER-1 | 5′‐CAGATAGATCCTAGTACAGAA‐3′ |
|  |  | | shFER-2  shFER-3  miR-206 inhibitor  inhibitor-NC | 5′‐AAAGAAATTTATGGCCCTGAG‐3′  5′‐AACTACGGTTGCTGGAGACAG‐3′  5′‐CCACACACUUCCUUACAUUCCA‐3′  5′‐CAGUACUUUUGUGUAGUACAA‐3′ |
