# Supplementary material for: FER Regulated by miR-206 Promotes Hepatocellular Carcinoma Progression via NF-κB Signaling
Source: Front Oncol. 2021 Jul 5;11:683878. doi: 10.3389/fonc.2021.683878 (PMC8289706; doi:10.3389/fonc.2021.683878)
Supplement: Supplementary file 2 [file Table_2.docx]

**Supporting Table 2**. Antibodies used in the present study.

| Protein | WB | IHC | Specificity | Product code |
| --- | --- | --- | --- | --- |
| FER | 1:2000 | 1:200 | Rabbit | Abcam, ab191060 |
| Ki67 |  | 1:200 | Rabbit | Abcam, ab16667 |
| E-cadherin  Vimentin | 1:1000  1:1000 | 1:400  1:200 | Rabbit  Rabbit | Cell Signaling Technology, #3195  Cell Signaling Technology, #5741 |
| NF-κB | 1:1000 |  | Rabbit | Abcam, ab32536 |
| p- NF-κB | 1:1000 |  | Rabbit | Cell Signaling Technology, #3033 |
| GAPDH | 1:1000 |  | Rabbit | Cell Signaling Technology, #5174 |
